# Morphology and function of pinniped necks: The long and short of it

**DOI:** 10.1002/ar.25642

**Published:** 2025-02-21

**Authors:** Justin Keller, Annalisa Berta, Mark Juhn, Blaire Van Valkenburgh

**Affiliations:** ^1^ Department of Ecology and Evolutionary Biology University of California Los Angeles California USA; ^2^ Department of Biology Chaffey College Rancho Cucamonga California USA; ^3^ Department of Biology San Diego State University San Diego California USA

**Keywords:** cervical vertebra, neck function, occipital area, Pinnipedia

## Abstract

Terrestrial vertebrates from at least 30 distinct lineages in both extinct and extant clades have returned to aquatic environments. With these transitions came numerous morphological adaptations to accommodate life in water. Relatively little attention has been paid to the cervical region when tracking this transition. In fully aquatic cetaceans, the cervical vertebrae are compressed, largely because a loss of neck mobility reduces drag. We ask whether this pattern of cervical evolution is present in the more recently evolved semiaquatic pinnipeds. Here, we compare neck morphology and function in three families of pinnipeds, the Otariidae, Phocidae, and Odobenidae as well as between pinnipeds and their terrestrial arctoid relatives (ursids and mustelids). Using cranial CT scans, we quantified the occipital surface area for neck muscle attachment as well as vertebral size and shape using linear measurements. Results show that pinnipeds have a relatively larger occipital surface area than ursids and terrestrial mustelids, suggesting that marine carnivorans have enlarged their neck muscles to assist with head stabilization during swimming. Within pinnipeds, we found quantitative differences in cervical morphology between otariids and phocids that coincide with their locomotor style. Phocids are hindlimb‐dominated swimmers that propel themselves with pelvic oscillations. Their necks are relatively stiff and their cervical vertebrae are compressed anteroposteriorly with reduced muscular attachment areas. By contrast, otariids are forelimb‐dominated swimmers that locomote in water and on land using their pectoral limbs, often recruiting their neck to initiate turns underwater as well as assisting in “walking” on land. Consequently, otariids have stronger, more flexible necks than phocids, which is reflected in more elongate cervical vertebral centra with larger muscle attachments. The walrus (Odobenidae) has a cervical vertebrae morphology intermediate to that of phocids and otariids, consistent with a phocid swimming mode combined with a more muscular neck that likely functions in intraspecific conflict and haul‐out behavior.

## 1 INTRODUCTION

The vertebrate transition from aquatic to terrestrial habitats required profound anatomical changes to accommodate life on land. Changes to the integument, development of lungs, and modification of the axial and appendicular skeleton were all essential for success in the new, much drier environment that also subjected them to increased gravitational forces. In response, tetrapods developed stronger limbs and larger vertebral centra that bore zygopophyses to aid in support of the spine on land. In addition, tetrapods evolved a specialized first cervical vertebra or atlas, that allowed limited head movements (Liem et al., 2001). Over time, additional anterior vertebra were modified, creating a neck and allowing more complex head movements (Arnold, 2021). Whereas locomotion in water favors stiff or no necks to minimize drag and increase stability when swimming (Fish, 1994), the relative ease of moving through air placed fewer limitations on neck evolution.

Despite the success of vertebrates on land, numerous groups have returned to the sea over the past 250 million years, including archosaurs, lepidosaurs, and mammals (Kelley & Pyenson, 2015; Uhen, 2007). Mammals have invaded the marine habitat at least seven times, as cetaceans, sirenians, and within three lineages of Carnivora—Ursidae ([polar bear], Mustelidae [otters], and Pinnipedia) (Otariidae [sea lions and fur seals], Phocidae [seals], and Odobenidae [walruses]), as well as extinct hippo‐like desmostylians (*Kolponomos* spp.), and an aquatic sloth (*Thalassocnus* spp.) (Uhen, 2007). The most aquatic of these groups, the cetaceans, shows extreme modifications for aquatic life such as complete loss of the hindlimbs, paddle‐like forelimbs, loss of hair, fusiform body shape, and a completely immobile, compressed, and often fused cervical vertebral series. The resultant lack of neck mobility reduces drag while swimming and mimics that of fishes, all of which lack necks. However, unlike the cetacean condition, pinnipeds retained flexible necks and thus must actively resist bending in this region to reduce drag. Consequently, we expect that pinnipeds will have increased musculature as evidenced by larger occiputs to stabilize the neck during swimming relative to their terrestrial relatives, such as ursids and mustelids (Park et al., 2024; Paterson et al., 2020). In addition, there are significant differences among pinnipeds in the degree of neck flexibility that should be reflected in vertebral form and strength. For example, otariids use an oscillatory motion in their forelimbs to generate propulsion while phocids and odobenids rely on undulation of their hindlimbs (Backhouse, 1961; English, 1976; Gordon, 1983; Tarasoff, 1972). The otariid swimming mode is associated with a strong and highly flexible neck, whereas the hindlimb‐dominated phocid mode is associated with a shorter, stiffer neck (Fish et al., 2003). In addition, otariids employ their neck when locomoting on land, swinging their head and neck laterally to assist their limbs (English, 1976; Kerr et al., 2018; Peterson & Bartholomew, 1967). The California sea lion (*Zalophus californianus*), the only otariid with movements examined in depth, is highly maneuverable and capable of a variety of acrobatic maneuvers, such as leaping from the water onto land (Fish et al., 2003: Leahy et al., 2021). This high degree of maneuverability is the result of increased flexibility of the axial skeleton (Leahy et al., 2021). Previous work (Buchholtz, 2001; Esteban et al., 2024; Long et al., 1997; Pierce et al., 2011) has shown that vertebral centrum dimensions strongly influence the range of motion at intervertebral joints, suggesting that phocid and otariid cervical centrum shape should differ in predictable ways. Relative to phocid and odobenid cervicals, otariid cervicals are predicted to be relatively larger with taller neural spines for attachment of comparatively large neck muscles. In addition, centrum shape is likely to differ, with otariids having more elongate centra as opposed to more compact centra in phocids. These swimming differences have also been related to ecology, for example, feeding behavior. Phocids employ their clawed forelimbs to help with their “hold and tear” feeding behavior, whereas otariids use a different strategy of “head shaking” at the water's surface when feeding on large prey (Hocking et al., 2016, 2017; Kienle et al., 2017).

## 2 MATERIALS AND METHODS

### 2.1 Occiput surface area

**FIGURE 1:**
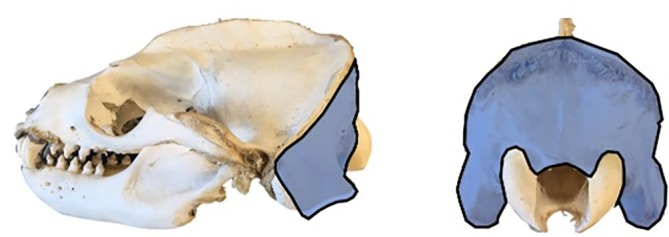
Visual representation of the surface area measurement for this study. Lateral (left) and posterior (right) views of a *Zalophus californianus* skull with occiput surface area represented in blue

Occipital surface area measurements were taken on CT scans of 35 skulls from 27 species of arctoid carnivorans (see Supporting Information, Table S1) using imaging program 3‐matic (v.7.01–13.0, Materialize). Surface area was defined as the area bounded dorsally by the nuchal margin of the occiput and laterally by the paraoccipital processes (Figure 1), excluding the occipital condyles. To investigate the effect of habitat, taxa were grouped into one of two categories, aquatic or terrestrial. Pinnipeds are usually considered to be semiaquatic, but for this study, we categorized species by where they forage; those that feed predominantly in water were defined as aquatic and those that feed predominantly on land were defined as terrestrial. Because of this, species such as the polar bear (*Ursus maritimus*) were defined as terrestrial in this study despite their propensity for swimming. Although there also are numerous nonpinniped examples of the evolution of semiaquatic habits within mammalian clades, such as rodents and shrews (see Hood, 2020), our occipital area study is focused on adaptations associated with the land‐to‐water transition within Carnivora.

**TABLE 1:** List of species used for occipital surface area and cervical vertebrae analyses, with species code, family membership, assigned habitat (aquatic or terrestrial), and the number and sex of the specimens.

Species	Code	Family	Habitat	Occiput N	Cervical vertebrae N
*Mephitis mephitis*	Mme	Mustelidae	Terrestrial	1 (f)	
*Mustela frenata*	Mfr	Mustelidae	Terrestrial	2 (1f,1m)	
*Neovison vison*	Nvi	Mustelidae	Terrestrial	1 (u)	
*Taxidea taxus*	Tta	Mustelidae	Terrestrial	2 (1f,1m)	
*Gulo gulo*	Ggu	Mustelidae	Terrestrial	2 (1f,1m)	
*Enhydra lutris*	Elu	Mustelidae	Aquatic	2 (1f,1m)	
*Lontra canadensis*	Lca	Mustelidae	Aquatic	2 (1f,1m)	
*Ailuropoda melanoleuca*	Ame	Ursidae	Terrestrial	1 (u)	
*Ursus americanus*	Uam	Ursidae	Terrestrial	1 (m)	
*U. arctos*	Uar	Ursidae	Terrestrial	2 (1f,1m)	
*U. maritimus*	Uma	Ursidae	Terrestrial	2 (2m)	
*Odobenus rosmarus*	Oro	Odobenidae	Aquatic	1 (u)	1 (u)
*Arctocephalus australis*	Aau	Otariidae	Aquatic	1 (u)	
*A. pusillus*	Apu	Otariidae	Aquatic	1 (u)	2 (1f,1m)
*A. townsendii*	Ato	Otariidae	Aquatic	1 (u)	
*Callorhinus ursinus*	Cur	Otariidae	Aquatic		1 (m)
*Emetopias jubatus*	Eju	Otariidae	Aquatic		3 (1m,2f)
*Halichoerus grypus*	Hgr	Phocidae	Aquatic	1 (m)	2 (1f,1m)
*Otaria byronia*	Oby	Otariidae	Aquatic	1 (f)	2 (1f,1m)
*Zalophus californianus*	Zca	Otariidae	Aquatic	2 (1f,1m)	4 (2f, 2m)
*Erignathus barbatus*	Eba	Phocidae	Aquatic	2 (1u,1f)	1 (m)
*Hydrurga leptonyx*	Hle	Phocidae	Aquatic	1 (f)	1 (f)^a^
*Leptonychotes weddelli*	Lwe	Phocidae	Aquatic		1 (f)
*Lobodon carcinophaga*	Lca	Phocidae	Aquatic		2 (f)
*Mirounga angustirostris*	Man	Phocidae	Aquatic	1 (f)	4 (2f,2m)
*M. leonina*	Mle	Phocidae	Aquatic	1 (m)	2 (1f,1m)
*Neomonachus schauinslandi*	Nsc	Phocidae	Aquatic		1 (f)
*N. tropicalis*	Ntr	Phocidae	Aquatic	1 (f)	
*Phoca fasciata*	Pfa	Phocidae	Aquatic	1 (u)	
*P. vitulina*	Pvi	Phocidae	Aquatic	1 (u)	3 (m)
*Pusa hispida*	Phi	Phocidae	Aquatic		1 (u)
*P. sibirica*	Psi	Phocidae	Aquatic		1 (f)

*Note*: A species code, family, sex (male, female, or unknown), and habitat (terrestrial or aquatic) are listed for each. For specimen museum ID numbers, see Supporting Information Table 1.

^a^ Only the atlas and axis were available for *H. leptonyx* (Leopard seal).

To examine the effect of body size on occiput surface area, we regressed occiput surface area on condylobasal skull length. Skull length was used as a proxy for body size given that specimen‐specific body mass data are quite difficult to obtain for marine mammals.

### 2.2 Cervical measurements

**FIGURE 2:**
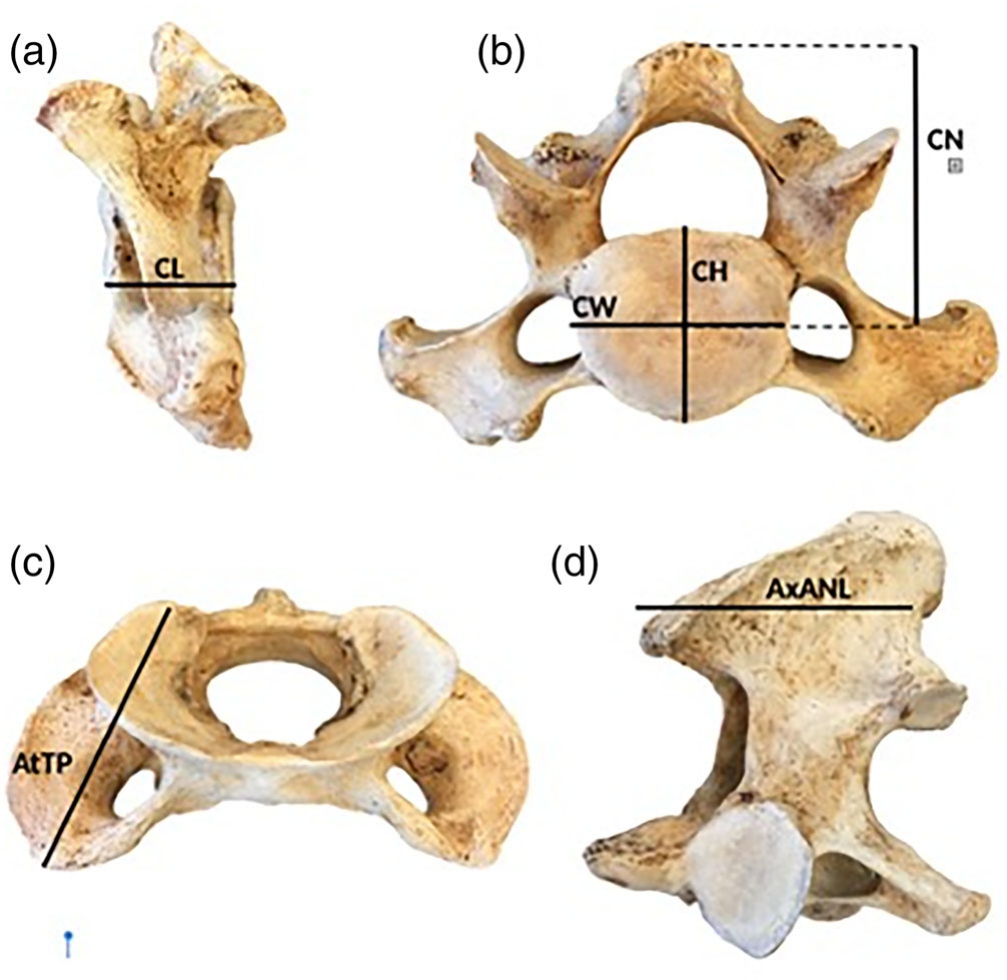
Visual representation of the linear measurements for this study. Cervical vertebrae of *Odobenus rosmarus*: (a) lateral view of C3 with centrum length (CL); (b) anterior view of C3 with centrum width (CW), centrum height (CH), and neural spine height (CNH); (c) anterior view of atlas with maximum lateral transverse process width (AtTT); (d) axis neural spine width (anteroposterior) (AxANL).

**FIGURE 3:**
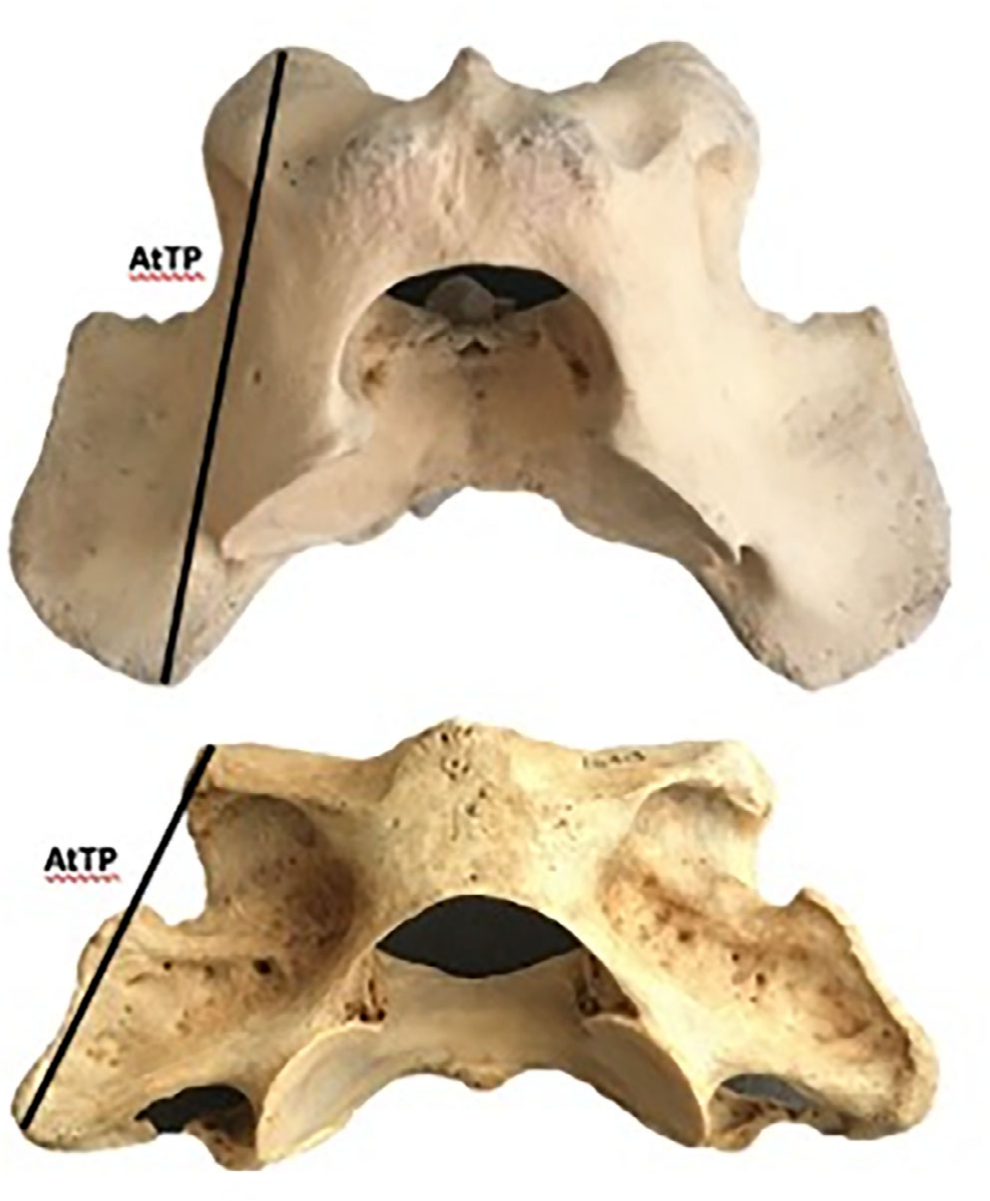
Dorsal view of two atlas vertebrae for an otariid (*E. jubatus*) (top) and phocid (*E. barbatus*) (bottom) showing the AtTP measurement used in this study. Note the expansion of the transverse process in the posterior direction in the otariid compared to the phocid.

The cervical vertebrae of 32 pinniped specimens representing 21 species (8 otariid, 12 phocid, and *Odobenus*) were measured from the collections of the Natural History Museum of Los Angeles County, the Museum of Vertebrate Zoology at the University of California, Berkeley, and the Donald R. Dickey Bird and Mammal Collection at the University of California, Los Angeles (Supporting Information, Table S2). The entire cervical series (C1–C7) was measured as well as condylobasal skull length. Cervical measurements were taken that approximated centrum dimensions and muscle attachment size. The linear measurements include centrum length (CL), height (CH), width (CW), and neural spine height (CNH) (Figure 2). Centrum plate area was estimated as the product of CH and CW. For each individual, we calculated the mean CL, CH, CW, and CNH, respectively, for the entire cervical series. Element‐specific measurements were also taken for the atlas (C1) and axis (C2) because the transverse processes of the atlas and neural spine of the axis are attachment sites for muscles involved in neck rotation and flexion. The element‐specific measurements were: maximum anteroposterior length of atlas transverse process (AtTP) and axis neural spine anteroposterior length (AxANL) (Figures 2 and 3). AtTP was measured instead of maximum mediolateral width of the transverse processes because the difference between the three pinniped families in this region is largely a posterior expansion of the process (Figure 3), and AtTP better captured that variation in shape.

Centrum plate area was compared with centrum length to approximate flexibility of the neck. Previous work on the thoracolumbar region shows that the relationship between centrum area and length impacts the degrees of freedom at each intervertebral joint (Jones & Pierce, 2016). Shorter centra with broader plate areas result in a less flexible neck than longer centra with smaller plate areas. CNH, AtTT, AtTP, and AxANL were each regressed against skull length to examine the effect of body size on vertebral dimensions within the three families.

### 2.3 Statistical analyses

We used least‐squares linear regression to examine allometric relationships between our skeletal measurements and skull length using the “lm” function in R (R Core Team, 2024). The expected slope value under isometry was two for squared parameters (occiput surface area and centrum plate area) versus skull length and one for linear measures (CNH, AtTT, AtTP, and AxANL) versus skull length. Slope comparisons between habitat groups and family were done using the R package “emmeans” (Lenth, 2024). In addition, to explore the impact of habitat (terrestrial vs. aquatic) and family (phocid vs. otariid) on our results, we employed multiple regression analysis using the “lm” function in R (R Core Team, 2024). None of the multiple regression analyses revealed any significant effects of either habitat (terrestrial vs. aquatic) in the occipital area comparisons or family membership (otariid vs. phocid) in the vertebral measurement analyses (see Supplementary Information, Regression Tables). Consequently, we include only the linear regression results below.

To test for phylogenetic signal in our results, we constructed a tree (Supporting Information, Figures S1 and S2) pruned from Upham et al. (2019) and calculated Blomberg's R and Pagel's λ using the R package “phytools” for the residuals from our previous multiple regressions (Revell, 2012). We found no phyletic signal in the residuals for Blomberg's R or Pagel's λ (see Supporting Information, Table S3). Therefore, there was no need to perform a phylogenetic generalized least‐squares (PGLS) to account for nonindependence of the species, and both simple and multiple regressions are appropriate for the dataset. Consequently, we include only the simple linear regression results below for ease of visualization.

## 3 RESULTS

### 3.1 Occiput surface area

**FIGURE 4:**
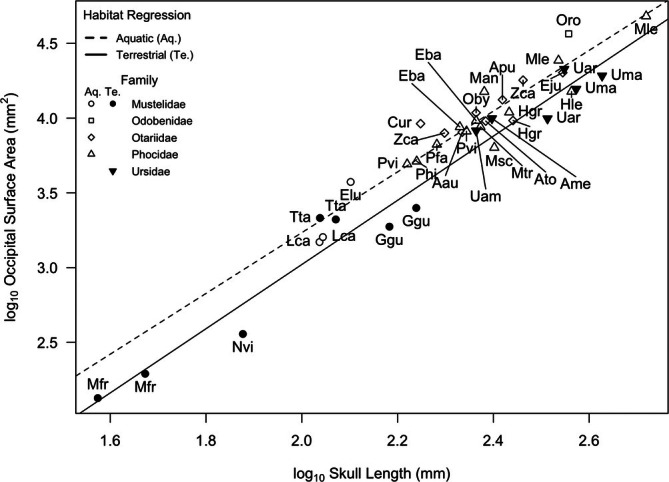
Scatterplot of occipital surface area versus skull length. Aquatic taxa (open symbols) and corresponding regression (dashed line), terrestrial taxa (filled symbols), and corresponding regression (solid line). Species labels from Table 1. See Table 2 for line equations and regression statistics.

Occiput surface area scaled similarly and isometrically with skull length in both aquatic and terrestrial taxa (*r* ^2^ ≥ 0.89) but the y‐intercept was larger in aquatic taxa (−0.83 vs. −1.27) (Figure 4, Table 2). However, this difference was not significant at the 0.05 level. Nevertheless, in general, pinnipeds have relatively larger occiput surface areas across all body sizes than their terrestrial relatives. There were exceptions to this pattern. Three phocids, the gray seal (Hgr), leopard seal (Hle), and Hawaiian monk seal (Nsc) had relatively reduced occipital areas and fell on the terrestrial regression line, and four arctoids, badgers (Tta), giant pandas (Ame), black bear (Uam), and male grizzly bear (Uar), had relatively broad occiputs and fell on the aquatic regression line. Among the pinnipeds, the walrus had an exceptionally large occiput which is consistent with its massive skull that functions in foraging in bottom sediments.

**TABLE 2:** Summary statistics from linear regressions used in this study.

Regression	*n*	Group	Slope	y‐intercept	*r* ^2^	CI for slope
Occiput SA vs. Skull Length	14	Terrestrial	2.15	−1.27^¨^	0.97**	1.88‐2.41
	26	Aquatic	2.03	−0.83^¨^	0.89**	1.73‐2.33
Centrum Area vs. Centrum Length	14	Otariidae	1.86	−0.51	0.94**	1,57‐2.14
	18	Phocidae	1.8	−0.27	0.98**	1.68‐1.92
Neural Spine Height vs. Skull Length	14	Otariidae	1.42*	−1.53^¨^	0.87**	1.08–1.76
	18	Phocidae	1.15*	−1.01^¨^	0.97**	1.05‐1.26
Atlas TP Length vs. Skull Length	14	Otariidae	1.30*	−1.25^¨^	0.97**	0.86‐1.74
	18	Phocidae	1.31*	−1.46^¨^	0.78**	1.2‐1.42
Axis NS Length vs. Skull Length	14	Otariidae	1.48*	−1.81¨	0.77**	0.98‐1.98
	18	Phocidae	1	−0.75^¨^	0.77**	0.73‐1.28

*Note*: That none of the differences in y‐intercept values for the pairwise comparisons achieved significance at the 0.05 level, perhaps as a result of insufficient sample size.

* Significant difference (*p* < 0.05) in slope from expected values of one (1).

** *p* < 0.05.

### 3.2 Cervical measurements

**FIGURE 5:**
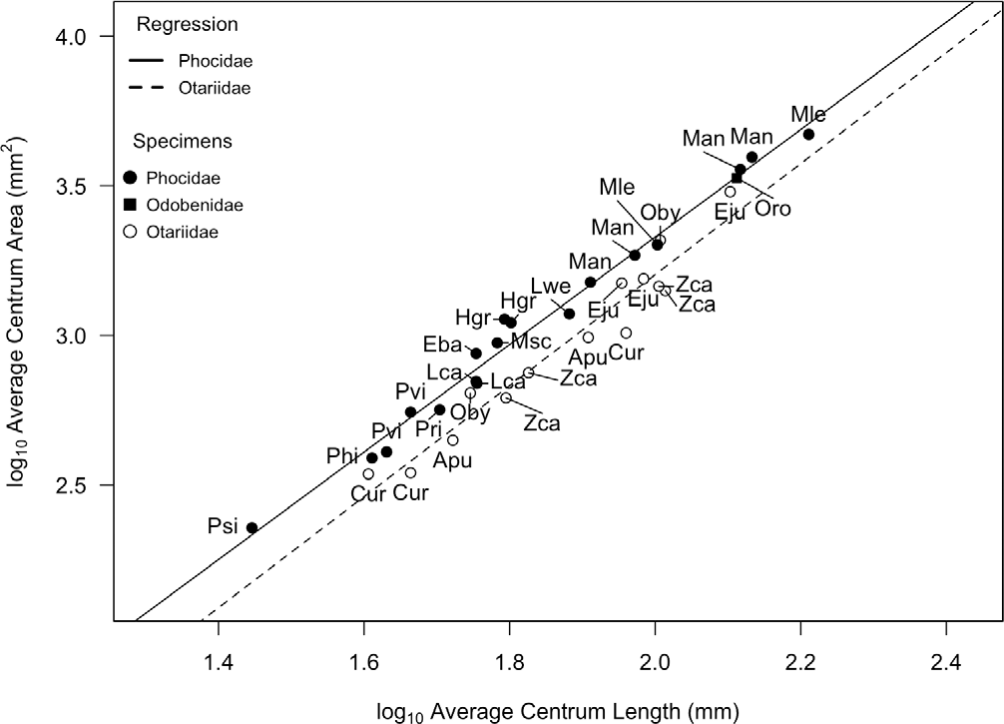
Scatterplot of average centrum area versus length within Pinnipedia. Centrum area values were averaged across C2‐C7 and log‐corrected to fit a linear model. Species labels from Table 1, 2. Regression lines: Otariidae (dashed). Phocidae with *Mirounga angustirostris* included (solid) and excluded (dotted). See Table 2 for line equations and regression statistics.

Cervical centrum plate area scaled with near isometry (slope = 1.8 for phocids, 1.86 for otariids) with centrum length in both otariids and phocids (Figure 5, Table 2). The y‐intercept is greater for the phocid than the otariid regression, indicating that phocid centrum plate area is larger than that of otariids across all centrum lengths. As expected, phocids have more compressed cervical vertebrae than similar‐sized otariids. The single walrus (Oro) fell on the phocid line, with an average centrum area close to that of the elephant seal (Man). Despite the strong correlations between centrum plate area and skull length (*r* ^2^ > 0.90), the observed difference in y‐intercept between the two families was not significant at the 0.05 level, perhaps due to our relatively small sample size.

**FIGURE 6:**
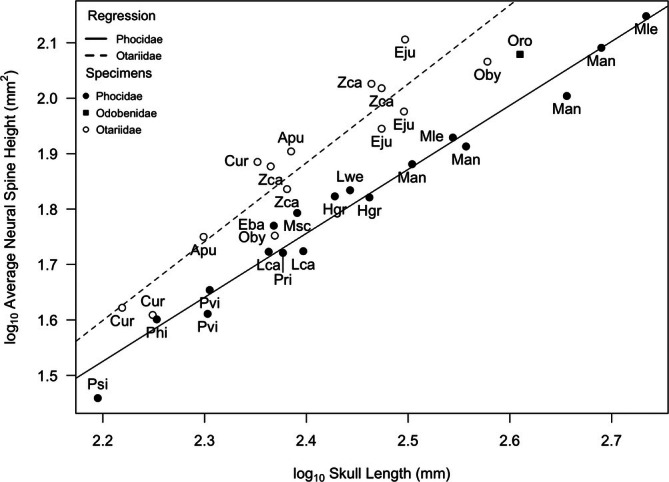
Log/log plot of average neural spine height (CNH) versus skull length within Pinnipedia. CNH values were averaged across C2‐C7 for each specimen and log‐corrected to fit a linear model. Species labels from Table 1, 2. Regression lines: Phocidae (solid) and Otariidae (dashed). Families are denoted by open circles (Otariidae) and filled circles (Phocidae). Lowercase letters after the species label denote sex (m: Male, f: Female, u: Undefined). See Table 2 for line equations and regression statistics.

Neural spine height (CNH) is positively allometric (*p* > 0.05) relative to skull length for both otariids and phocids (Figure 6, Table 2). In both families, large species have proportionally longer neural spines than smaller species. The slope was more positive in otariids than phocids, but the difference was only marginally significant (*p* = 0.08), and CNH tended to be larger in otariids than phocids across all skull lengths. There were two exceptions to this pattern, however, among the otariids, both reflecting sexual dimorphism. The female South American sea lion (Oby) as well as the female northern fur seal (Cur) fell close to the phocid regression line. The walrus (Oro) fell in between the phocid and otariid lines and close to the male South American sea lion (Oby).

**FIGURE 7:**
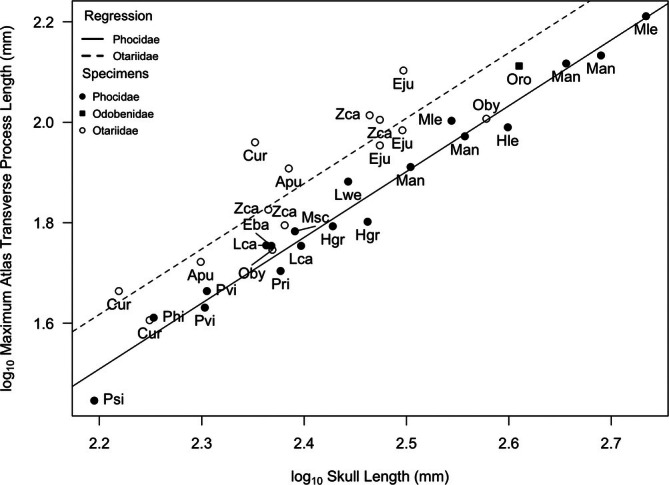
Log/log plot of maximum atlas transverse process length (AtTP) against skull length within Pinnipedia. Values log_10_ transformed to fit a linear model. Species labels from Table 1, 2. Regression lines: Phocidae (solid) and Otariidae (dashed). See Table 2 for line equations and regression statistics.

Atlas transverse process length (AtTP) scaled with positive allometry (*p* < 0.05, slope = 1.3) and similarly in both otariids and phocids (Figure 7, Table 2), indicating that larger individuals have proportionally larger AtTP than smaller taxa in both families. As with CNH, the walrus fell in between the two lines and exceeded phocids of similar size (elephant seals [Man]). Notably, AtTP is almost always larger in otariids, relative to phocids across all body sizes as reflected by the higher y‐intercept of the otariid regression line relative to that of phocids (Table 2). There were four exceptions to this pattern among the otariids: two females (California sea lion [Zca] and northern fur seal [Cur]), and both male and female South American sea lions (Oby), all of which fell close to the phocid regression line with relatively reduced AtTP. Again, the walrus (Oro) was intermediate in morphology, falling between the otariid and phocid regression lines.

**FIGURE 8:**
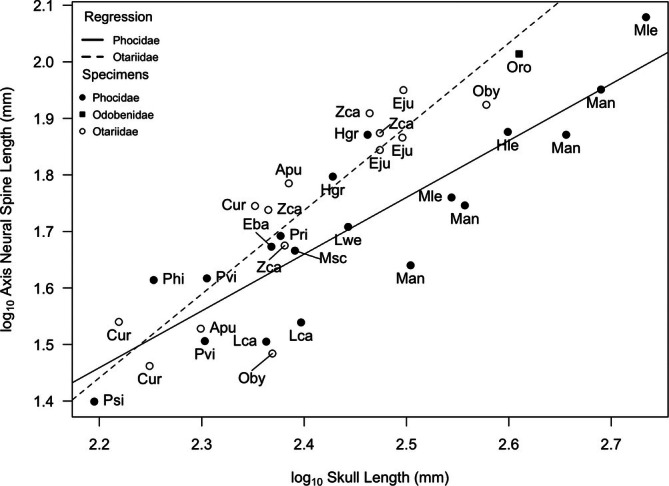
Log/log plot of anteroposterior neural spine length of the axis (AxANL) against skull length within Pinnipedia. Values log_10_ transformed to fit a linear model. Species labels from Table 1 Regression lines: Phocidae (solid) and Otariidae (dashed). See Table 2 for line equations and regression statistics.

The anteroposterior neural spine length of the axis (AxANL) scaled with significant positive allometry (*p* = 0.04, slope = 1.48) in otariids and isometrically (slope = 1) in phocids (Figure 8, Table 2), indicating that larger otariids tend to have longer neural spines. The walrus (Oro) also had a long‐axis neural spine and plotted close to the otariid line. Although the otariids had relatively longer‐axis neural spines than most phocids, there was overlap in neural spine height between phocids and otariids at smaller body size (e.g., Northern fur seal [Cur] and two phocids, harbor seal [Pvi], and ringed seal [Phi]).

## 4 DISCUSSION

With few exceptions, the pinnipeds had larger occiputs for their skull length than their putative ancestors, terrestrial arctoids, including ursids and mustelids. This is consistent with pinnipeds having larger areas of attachment for muscles that control head movement and is likely a response to increased drag experienced by the animals moving forward in water as opposed to air. Holding the head in line with the body as they move forward allows them to maintain their streamlined shape and minimize drag. Whereas fully aquatic cetaceans have solved this problem through extreme reductions in neck flexibility (Goel et al., 2021; Marchesi et al., 2020), pinnipeds retain a supple neck and consequently evolved a more muscular neck to control head movement in a fluid medium.

The association between greater occiput area and aquatic habits is also apparent with other aquatic arctoids, although to a lesser degree. Sea otters (*Enhydra lutris*) have a larger occiput area compared with the other sampled mustelids, suggesting that they also have evolved greater musculature for controlling head movements in water. Interestingly, fossorial badgers (*Taxidea taxus*) have a somewhat large occiput relative to their skull length, which might reflect the fact that they have relatively small skulls rather than stronger necks. However, our sample size of mustelid species (*n* = 7) is small and our results should be confirmed with a larger sample.

Although the three pinniped families were similar in relative occiput area, they differed greatly in aspects of their cervical vertebral morphology that have functional significance. For example, phocids and our single walrus have centra that are short anteroposteriorly and broad mediolaterally and dorsoventrally (large centrum plate surface area), resulting in a disc‐shape as opposed to the more elongate spool‐shape of otariid centra (Esteban et al., 2024). More disc‐like centra with large centrum plate areas have fewer degrees of freedom at each vertebral joint (Long et al., 1997; Pierce et al., 2011), resulting in less overall neck flexibility. The reduced centrum area in otariids relative to phocids and the walrus is likely driven by differences in their locomotor styles. In the otariid California sea lion (*Z. californianus*), propulsion is generated by the forelimbs as the neck initiates a turn by bending dorsally (Fish et al., 2003). The increased flexibility along the cervical spine allows a more streamlined shape while the neck is bent and aids in conserving velocity lost during the turn. Sea lions also have increased flexibility across the entire spine, which further aids in conservation of velocity (Fish et al., 2003; Godfrey, 1985).

In contrast, phocids, including the leopard seal, a species with an otariid‐like foreflipper (Hocking et al., 2021) and walruses, generate propulsion from their hindlimbs, maintaining a relatively rigid neck during locomotion while using their forelimbs to aid in stability (Fish et al., 1988). Phocids generate thrust using flippers that are posterior to the body's center of gravity. This arrangement (as opposed to the forelimb propulsion of otariids) is highly stable and allows phocids to pitch, yaw, and roll with ease as they swim through the water column (Fish, 2002).

AtTP, AxANL, and CNH reflect attachment sites for a number of muscles that control movement of the head relative to the body. AtTP approximates the attachment of the *m. obliquus capitis cranialis* on the ventral side and the *m. obliquus capitis caudalis* on the dorsal side of the transverse process of the atlas (Antón & Galobart, 1999). These two muscles are involved in head dorsiflexion and ventroflexion, respectively, as well as lateral flexion of the head when the muscle masses on the left or right side contract in isolation. AxANL approximates the attachment area of the *m. obliquus capitis caudalis* (lateral surface) and *m. rectus capitus* (dorsal border of spine) on the axis. These muscles are involved in neck rotation and dorsiflexion, respectively (Antón & Galobart, 1999). CNH approximates the attachment area for the *m. multifidus* on C2‐C7, another muscle involved in neck dorsiflexion.

Not surprisingly, the more flexible‐necked otariids had larger transverse processes (AtTP) and neural spines (CNH) than the shorter‐necked phocids. A more flexible neck requires larger muscles for the control of movements while swimming. Specifically, the fact that the transverse processes of the atlas (AtTP) extend more posteriorly in otariids than phocids would not only increase muscle attachment area but also increase the resting length of the *m. obliquus capitis cranialis* and *m. obliquus capitis caudalis*, because they originate farther from the insertion point on the skull. The larger muscle attachment areas of otariid cervicals relative to those of phocids likely result in stronger necks and therefore greater control and stability of the neck in every direction (dorsiflexion, ventroflexion, and rotation). Moreover, the combined strength and mobility of the otariid head and neck allow these mammals to turn rapidly when needed, as well as use these muscle groups in concert to maintain a streamlined shape while cruising.

The walrus appears to have a cervical vertebrae morphology that is intermediate to the other two pinniped families. The walrus has a massive skull with a relatively large occipital surface area (Figure 4) suggesting substantial neck musculature. Its discoidal cervical vertebrae are shaped more like those of stiff neck phocids than flexible neck otariids, which is consistent with their reliance on hindlimb propulsion. In addition, Esteban et al. (2024) suggested that the stiff neck of the walrus could reflect their habit of hauling themselves onto the ice using their tusks, as well as their tendency to feed on small sessile invertebrates on the seafloor. However, the relative length of their neural spines, both across the entire cervical series (Figure 6) and those of the atlas and axis (Figures 7 and 8), were either intermediate in size to phocids and otariids, or in the case of axis neural height (AxN) more similar to that of otariids. Given that we were only able to include a single individual in our study, our conclusions are tentative, but we suggest that the walrus has relatively more musculature for head dorsiflexion and rotation than phocids, likely because walruses (male and female) use their weighty heads and tusks in intraspecific combat, and also as mentioned above, for hauling out on sea ice.

At least two factors likely influence differences in neck flexibility within and between phocids, otariids, and the walrus. The first is habitat complexity of the environments in which they forage, and the second is how they locomote on land. Chasing a prey item in a complex environment, such as a kelp forest or tropical reef, requires agility. Prey frequently dart sideways to retreat into complex habitat for cover. Studies of the otariid, *Zalophus*, suggest that their extreme neck flexibility represents an adaptation to shallow complex foraging habitats (e.g., kelp forests) as opposed to less complex, off‐shore pelagic habitats (Fish et al., 2003). Whales have completely immobile necks, restricting them to pursuing prey in a relatively straight line. However, the Amazon river dolphin (*Inia geoffrensis*) is an exception among cetaceans. River dolphins forage in shallow habitats with high complexity, such as flooded forests or rivers, and maintain some flexibility in their neck to aid in catching prey that avoid them with rapid, evasive turns (Fish, 2002). Across cetaceans, postcervical centrum shape was associated with habitat (Gillet et al., 2019, 2022; Marchesi et al., 2022). Pelagic species have more disc‐shaped centra, with coastal species having spool‐shaped centra. The correlation between increased neck flexibility and greater habitat complexity in both pinnipeds and cetaceans suggests strong and similar selection pressures on foraging ability on both groups. However, it does not seem to explain the lack of flexibility in phocid necks, as many of them overlap with otariids in geographic range and presumed foraging habitats, but details of their neck mobility during foraging behavior are lacking.

As noted above, the contrast between phocid, otariids, and odobenids in neck morphology might also reflect differences in how they locomote on land. Compared with phocids, otariids are less specialized for aquatic life and employ all four limbs to move on land (sometimes rapidly) while using their necks to hold their heads well above the substrate. Phocids cannot rotate their hindlimbs under their body and instead move by rapid dorsoventral undulations of their whole body, or galumphing, with their head in line with their body and close to the ground. The walrus is again intermediate; similar to the otariids, it uses all four limbs to walk on land, but unlike the otariids, the anterior limbs of the walrus function primarily to lift the body off the surface so that the spine and hindlimbs can thrust the body forward (Gordon, 1981), in an action that is more akin to that of phocids.

The locomotor differences between phocids and otariids both on land and in water suggest that the neck is playing a different functional role in each group. The more aquatic phocids have less flexible necks because of their increased reliance on aquatic life relative to pinnipeds. The stark contrast between the two pinniped families suggests that they have solved the problem of mitigating drag at the leading edge of their bodies in two alternative ways. Reduced flexibility allows phocids to passively maintain rigidity in a fluid medium but likely limits their turning speed and agility. Otariids evolved in a different direction, in which neck mobility and strength were maintained or even enhanced, allowing for supple, swift movements, but at a cost of maintaining enlarged cervical muscles and vertebrae (see Esteban et al., 2024). It is possible that the energetic cost of swimming is greater in otariids than phocids, but this has yet to be documented. Limited data (i.e., California sea lions and harbor seals) suggest that regardless of swimming style the energetic costs are similar among otariids and phocids (Williams, 1999). As a forager of relatively immobile prey, the walrus has no need for rapid turns and a highly flexible neck, although their necks do swing left and right as they move on land, in tandem with the raising and lowering of their forelimbs (Gordon, 1981).

It may be that otariids and phocids took these alternative evolutionary paths to reduce competition and increase ecological separation. A number of studies have looked at the relationship between diet and morphology in pinnipeds, but direct ecomorphological inferences have been difficult, in part due to extensive morphological convergence among phocids (Adam & Berta, 2002; Kienle et al., 2018). Moreover, some pinniped species will alter their feeding behavior depending on the foraging environment (Hocking et al., 2014), as well as the behavior of the prey (Bowen et al., 2002). Available data suggest that both sympatric phocids and sympatric otariids have divergent foraging and habitat preferences (Valenzuela‐Toro et al., 2023, 2024). Robust investigations into the role habitat complexity plays in neck evolution would require data on exact foraging distance from shore and depth in the water column. Spatial distribution data alone are not sufficient as otariids and phocids have a large degree in overlap in their occurrence data given that both families reproduce on land. To better understand the functional significance of neck morphology in phocids and otariids, we need more detailed data on dietary and foraging differences, as well as quantitative estimates of top speed, agility, and costs of locomotion on land and in water. Once we tease apart the ecological factors that drive the observed neck flexibility in extant groups, then we can investigate the feeding and locomotor ecology of extinct groups. If robust correlates between ecology and morphology are established, inferences based on specimens that are highly damaged or incomplete, common in the fossil record, can be more readily made. We look forward to revisiting the question of neck variation in pinnipeds in light of these new data.

## AUTHOR CONTRIBUTIONS

**Justin Keller:** Conceptualization; investigation; writing – original draft; methodology; writing – review and editing; formal analysis; visualization. **Annalisa Berta:** Writing – review and editing; validation; investigation. **Mark Juhn**: Formal analysis; writing‐review and editing. **Blaire Van Valkenburgh:** Supervision; writing – review and editing; conceptualization; methodology; project administration; visualization; investigation; validation.

## Supporting information


**Data S1.** Supporting Information.
