# Supplementary material for: Morphology and function of pinniped necks: The long and short of it
Source: Anat Rec (Hoboken). 2025 Feb 21;308(12):3175–85. doi: 10.1002/ar.25642 (PMC12594188; doi:10.1002/ar.25642)
Supplement: Supplementary file 1 — Data S1. Supporting Information. [file AR-308-3175-s001.docx]

**SUPPORTING INFORMATION (Keller et al.)**

**Supporting Information -Table 1**. Specimens and measurement data (all in mm) for the relative occipital area analyses. SKULL L, condylobasal skull length; OCCIPUT SA, estimated surface area of the occipital plate.

| Collection^1^ | ID NUM | SPECIES | FAMILY | SEX | HABITAT | SKULL L | OCCIPUT SA |
| --- | --- | --- | --- | --- | --- | --- | --- |
| USFW |  | Enhydra_lutris | Lutrinae | F | Aquatic | 126.53 | 3739.56 |
| UCLA | 18958 | Lontra_canadensis | Lutrinae | F | Aquatic | 108.99 | 1481.5 |
| UCLA | 15275 | Lontra_canadensis | Lutrinae | M | Aquatic | 110.6 | 1598.05 |
| UCLA | 15306 | Odobenus_rosmarus | Odobenidae | U | Aquatic | 360.81 | 36621.03 |
| LACM | 97270 | Otaria_byronia | Otariidae | F | Aquatic | 231.33 | 10854.26 |
| LACM | 52235 | Arctocephalus_pusillus | Otariidae | U | Aquatic | 262.17 | 13265.71 |
| LACM | 84079 | Halichoerus_grypus | Otariidae | M | Aquatic | 276.28 | 9636.82 |
| LACM | 95730 | Zalophus_californianus | Otariidae | F | Aquatic | 198.53 | 7943.73 |
| UCLA | 252 | Zalophus_californianus | Otariidae | M | Aquatic | 289.96 | 17928.43 |
| UCLA | 260 | Arctocephalus_townsendi | Otariidae | U | Aquatic | 242.27 | 9552.82 |
| LACM | 31697 | Arctocephalus_australis | Otariidae | U | Aquatic | 216.08 | 8046.27 |
| UCLA | 123458 | Phoca_vitulina | Phocidae | U | Aquatic | 220.91 | 8173.91 |
| USNM | 269533 | Hydrurga_leptonyx | Phocidae | F | Aquatic | 365.74 | 15082.37 |
| LACM | 97254 | Mirounga_angustirostris | Phocidae | F | Aquatic | 240.47 | 15016.33 |
| USNM | 102527 | Neomonachus_tropicalis | Phocidae | F | Aquatic | 235.81 | 8755.4 |
| LACM | 52353 | Erignathus_barbatus | Phocidae | U | Aquatic | 231.08 | 9609.22 |
| LACM | 72574 | Erignathus_barbatus | Phocidae | F | Aquatic | 213.87 | 8734.39 |
| LACM | 84290 | Mirounga_leonina | Phocidae | M | Aquatic | 524.09 | 48085.81 |
| LACM | 52324 | Histriophoca_fasciata | Phocidae | U | Aquatic | 191.42 | 6663.5 |
| LACM | 30292 | Phoca_vitulina | Phocidae | U | Aquatic | 165.87 | 4925.48 |
| LACM | 52312 | Eumetopias_jubatus | Otariidae | F | Aquatic | 350.92 | 20136.71 |
| LACM | 97584 | Halichoerus_grypus | Phocidae | F | Aquatic | 271.29 | 10945.26 |
| LACM | 52355 | Neomonachus_schauinslandi | Phocidae | F | Aquatic | 252.06 | 6359.72 |
| LACM | 84245 | Mirounga_leonina | Phocidae | F | Aquatic | 343.37 | 24311.46 |
| LACM | 96757 | Pusa_hispida | Phocidae | U | Aquatic | 173.62 | 5142.74 |
| LACM | 51353 | Callorhinus_ursinus | Otariidae | F | Aquatic | 177.17 | 9173.08 |
| USNM | 95054 | Mustela_frenata | Mustelidae | F | Terrestrial | 37.51 | 134.09 |
| USNM | 52702 | Mustela_frenata | Mustelidae | M | Terrestrial | 47.08 | 195 |
| UCLA | 8488 | Neogale_vison | Mustelidae | U | Terrestrial | 75.35 | 359.98 |
| LACM | 45012 | Taxidea_taxus | Mustelidae | F | Terrestrial | 117.77 | 2099.79 |
| UCLA | 1481 | Taxidea_taxus | Mustelidae | M | Terrestrial | 109.18 | 2149.81 |
| USNM | 14885 | Gulo_gulo | Mustelidae | M | Terrestrial | 173.36 | 2505.43 |
| USNM | 157327 | Gulo_gulo | Mustelidae | F | Terrestrial | 152.5 | 1879.08 |
| USNM | 6072 | Ailuropoda_melanoleuca | Ursidae | U | Terrestrial | 249.24 | 9951.52 |
| USNM | 227070 | Ursus_americanus | Ursidae | M | Terrestrial | 231.46 | 8237.36 |
| USNM | 98062 | Ursus_arctos | Ursidae | F | Terrestrial | 325.7 | 9922.49 |
| ISM | H001-05 | Ursus_maritimus | Ursidae | M | Terrestrial | 374.41 | 15657.23 |
| LACM | 11911 | Ursus_maritimus | Ursidae | M | Terrestrial | 424.05 | 19202.64 |
| LACM | 92310 | Ursus arctos | Ursidae | M | Terrestrial | 352.8 | 21287.24 |

^1^Collections: USFW, United States Fish and Wildlife Service; UCLA, D.R. Dickey Bird and Mammal Collection; LACM, Natural History Museum of Los Angeles^:^ County; MVZ, Museum of Vertebrate Zoology, University of California, Berkeley; USNM, United States national Museum, Washington, D.C.; ISM, Illinois State Museum, Springfield, IL.

**Supporting Information-Table 2.** Specimens and measurement data (all in mm) for the cervical vertebra analyses. “NA” indicates that a measurement could not be taken. SKULL L, condylobasal skull length; AVG C AREA, average surface area of the vertebral centra; AVG C LENGTH, average anterior-posterior length of the vertebral centra; AVG C NH, average height of the neural spines across all centra; AxANL, axis neural spine anteroposterior length; AtTP, maximum anteroposterior length of atlas transverse process.

| Collection^1^ | ID NUM | SPECIES | | FAMILY | SEX | SKULL L | AVG C AREA | AVG C  LENGTH | AVG C NH | AxANL | AtTP |
| --- | --- | --- | --- | --- | --- | --- | --- | --- | --- | --- | --- |
| UCLA | 15306 | | Odobenus_rosmarus | Odobenidae | M | 407.81 | 3355.05 | 49.62 | 120.01 | 103.38 | 129.40 |
| LACM | 52358 | | Arctocephalus_pusillus | Otariidae | M | 242.47 | 984.55 | 37.57 | 80.17 | 61.02 | 80.99 |
| LACM | 52359 | | Arctocephalus_pusillus | Otariidae | F | 198.95 | 446.01 | 31.04 | 56.23 | 33.71 | 52.70 |
| LACM | 51357 | | Callorhinus_ursinus | Otariidae | M | 224.77 | 1019.72 | 51.52 | 76.81 | 55.59 | 91.16 |
| LACM | 52313 | | Eumetopias_jubatus | Otariidae | F | 297.58 | 1495.75 | 55.86 | 88.02 | 69.86 | 89.85 |
| LACM | 91336 | | Otaria_byronia | Otariidae | M | 378.65 | 2080.16 | 59.78 | 116.50 | 83.92 | 101.69 |
| LACM | 97270 | | Otaria_byronia | Otariidae | F | 233.82 | 641.51 | 31.35 | 56.48 | 30.51 | 55.70 |
| LACM | 343 | | Zalophus_californianus | Otariidae | M | 291.15 | 1408.89 | 64.13 | 106.11 | 81.02 | 103.27 |
| LACM | 39653 | | Zalophus_californianus | Otariidae | M | 298.08 | 1458.58 | 62.75 | 104.23 | 74.74 | 101.18 |
| LACM | 51234 | | Zalophus_californianus | Otariidae | F | 240.22 | 617.83 | 42.49 | 68.60 | 47.34 | 62.34 |
| LACM | 91889 | | Zalophus_californianus | Otariidae | F | 231.62 | 749.70 | 44.27 | 75.29 | 54.67 | 66.97 |
| LACM | 51353 | | Callorhinus_ursinus | Otariidae | F | 177.53 | 343.29 | 23.71 | 40.62 | 29.00 | 40.39 |
| LACM | 51351 | | Callorhinus_ursinus | Otariidae | F | 165.45 | 347.03 | 24.32 | 41.90 | 34.65 | 46.16 |
| UCLA | 14394 | | Eumetopias_jubatus | Otariidae | F | 313.14 | 1545.18 | 60.40 | 94.66 | 73.53 | 96.48 |
| UCLA | 14393 | | Eumetopias_jubatus | Otariidae | M | 314.22 | 3016.64 | 68.80 | 127.66 | 89.04 | 126.85 |
| MVZ | 127753 | | Hydrurga_leptonyx | Phocidae | U | 396.74 | NA | NA | NA | 75.11 | 97.62 |
| MVZ | 127755 | | Leptonychotes_weddellii | Phocidae | F | 277.39 | 1180.07 | 39.92 | 68.17 | 51.09 | 76.29 |
| MVZ | 127752 | | Lobodon_carcinophaga | Phocidae | F | 230.84 | 689.51 | 27.78 | 52.84 | 32.01 | 56.93 |
| MVZ | 127751 | | Lobodon_carcinophaga | Phocidae | F | 249.50 | 702.23 | 28.05 | 52.93 | 34.63 | 56.78 |
| LACM | 84245 | | Mirounga_leonina | Phocidae | F | 349.55 | 2010.24 | 39.32 | 84.90 | 57.61 | 100.66 |
| LACM | 84290 | | Mirounga_leonina | Phocidae | M | 541.86 | 4698.79 | 63.47 | 140.51 | 119.97 | 162.61 |
| LACM | 52355 | | Neomonachus_schauinslandi | Phocidae | F | 246.07 | 944.90 | 35.44 | 62.10 | 46.34 | 60.65 |
| LACM | 84272 | | Phoca_vitulina | Phocidae | M | 201.10 | 407.85 | 24.77 | 40.87 | 32.05 | 42.78 |
| LACM | 85993 | | Phoca_vitulina | Phocidae | M | 202.02 | 553.25 | 28.72 | 45.13 | 41.44 | 46.18 |
| LACM | 96757 | | Pusa_hispida | Phocidae | NA | 179.08 | 388.88 | 28.54 | 39.87 | 41.16 | 40.82 |
| MVZ | 140629 | | Pusa_sibirica | Phocidae | F | 156.74 | 226.83 | 19.10 | 28.75 | 25.07 | 27.95 |
| UCLA | 16413 | | Erignathus_barbatus | Phocidae | M | 233.60 | 868.15 | 35.06 | 58.86 | 47.13 | 56.74 |
| LACM | 84079 | | Halichoerus_grypus | Phocidae | M | 289.87 | 1100.79 | 40.00 | 66.26 | 74.37 | 63.39 |
| LACM | 97584 | | Halichoerus_grypus | Phocidae | F | 267.78 | 1133.19 | 41.87 | 66.54 | 62.61 | 62.05 |
| LACM | 54764 | | Mirounga_angustirostris | Phocidae | F | 319.21 | 1505.65 | 35.23 | 76.03 | 43.69 | 81.53 |
| LACM | 54765 | | Mirounga_angustirostris | Phocidae | M | 452.91 | 3587.33 | 49.69 | 100.95 | 74.28 | 130.80 |
| LACM | 54787 | | Mirounga_angustirostris | Phocidae | M | 490.27 | 3947.82 | 63.58 | 123.29 | 89.42 | 135.77 |
| LACM | 97254 | | Mirounga_angustirostris | Phocidae | F | 360.34 | 1853.60 | 42.94 | 81.84 | 55.77 | 93.83 |
| UCLA | 14402 | | Phoca vitulina richardii_ | Phocidae | M | 238.22 | 563.57 | 30.95 | 52.60 | 49.24 | 50.57 |

^1^Collections: UCLA, D.R. Dickey Bird and mammal Collection; LACM, Natural History Museum of Los Angeles^:^ County; MVZ, Museum of Vertebrate Zoology, University of California, Berkeley.

**Supporting Information : Regression Results**

The key variable in the tables for the multiple regression are the interactions, e.g. SKL : Habitat for the first table. This syntax refers to the interaction term of Skull Length and Habitat. If the probability of the t-statistic (i.e. *p-*value for that coefficient) is > 0.5, then there is no difference in the slope between both habitat types. TER refers to terrestrial. Since Habitat is categorial, there is a standard that the variable is compared to. In this case, the table is giving the coefficient estimate when the variable of Habitat is terrestrial.

**Occiput Surface Area**

**Occiput Surface Area** as a function of **Skull Length** and **Habitat Type**

| Variables | Estimate | Std. Error | t-Statistic | Prob | 2.5% | 97.5% |
| --- | --- | --- | --- | --- | --- | --- |
| Intercept | -0.8313 | 0.3735 | -2.226 | 0.0326 | -1.5896 | -0.0731 |
| SKL | 2.0326 | 0.1578 | 12.884 | 7.61e-15 | 1.7123 | 2.3528 |
| Habitat (TER) | -0.4426 | 0.4409 | -1.004 | 0.3224 | -1.3377 | 0.4525 |
| SKL:Habitat | 0.1147 | 0.1895 | 0.605 | 0.5489 | -0.2700 | 0.4995 |
|  | | | | | | |
| N | 39 | Residual SE | 0.1254 |  |  |  |
| Multiple R^2^ | 0.954 | Adjusted R^2^ | 0.95 |  |  |  |
| F-Statistic | 241.9 | *p-*value | < 2.2e-16 |  |  |  |

Slopes for Terrestrial and Aquatic taxa are not significantly different (*p* = 0.5489)

**Occiput Surface Area** as a function of **Skull Length**, only **Terrestrial**

| Variables | Estimate | Std. Error | t-Statistic | Prob | 2.5% | 97.5% |
| --- | --- | --- | --- | --- | --- | --- |
| Intercept | -1.2739 | 0.2676 | -4.76 | 0.00059 | -1.8630 | -0.6849 |
| SKL | 2.1473 | 0.1200 | 17.90 | 1.75e-09 | 1.8832 | 2.4114 |
|  | | | | | | |
| N | 13 | Residual SE | 0.1432 |  |  |  |
| Multiple R^2^ | 0.9668 | Adjusted R^2^ | 0.9638 |  |  |  |
| F-Statistic | 320.3 | *p-*value | 1.755e-09 |  |  |  |

**Occiput Surface Area** as a function of **Skull Length**, only **Aquatic**

| Variables | Estimate | Std. Error | t-Statistic | Prob | 2.5% | 97.5% |
| --- | --- | --- | --- | --- | --- | --- |
| Intercept | -0.8313 | 0.3465 | -2.399 | 0.0245 | -1.5465 | -0.1162 |
| SKL | 2.0326 | 0.1464 | 13.888 | 5.73e-13 | 1.7305 | 2.3346 |
|  | | | | | | |
| N | 26 | Residual SE | 0.1163 |  |  |  |
| Multiple R^2^ | 0.8893 | Adjusted R^2^ | 0.8847 |  |  |  |
| F-Statistic | 192.9 | *p-*value | 5.734e-13 |  |  |  |

**Centrum Area**

**Centrum Area** as a function of **Centrum Length** and **Family**

| Variables | Estimate | Std. Error | t-Statistic | Prob | 2.5% | 97.5% |
| --- | --- | --- | --- | --- | --- | --- |
| Intercept | -0.5104 | 0.2016 | -2.531 | 0.0173 | -0.9235 | -0.0974 |
| C Length | 1.8567 | 0.1070 | 17.346 | <2e-16 | 1.6374 | 2.0760 |
| Family (Pho) | 0.2438 | 0.2401 | 1.015 | 0.3186 | -0.2481 | 0.7357 |
| C Length : Family | -0.0591 | 0.1284 | -0.460 | 0.6488 | -0.3221 | 0.2039 |
|  |  |  |  |  |  |  |
| N | 32 | Residual SE | 0.0584 |  |  |  |
| Multiple R^2^ | 0.9713 | Adjusted R^2^ | 0.9683 |  |  |  |
| F-Statistic | 316.2 | *p-*value | < 2.2e-16 |  |  |  |

Slopes for Phocidae and Otariidae are not significantly different (*p* = 0.6488)

Note: *Hydrurga leptonyx* (Phocidae) does not have a Centrum Area or Centrum Length – making N = 32.

**Centrum Area** as a function of **Centrum Length**, only **Phocidae**

| Variables | Estimate | Std. Error | t-Statistic | Prob | 2.5% | 97.5% |
| --- | --- | --- | --- | --- | --- | --- |
| Intercept | -0.2666 | 0.1052 | -2.535 | 0.0221 | -0.4896 | -0.0437 |
| C Length | 1.7976 | 0.0572 | 31.440 | 8.2e-16 | 1.6764 | 1.9188 |
|  | | | | | | |
| N | 18 | Residual SE | 0.0471 |  |  |  |
| Multiple R^2^ | 0.9841 | Adjusted R^2^ | 0.9831 |  |  |  |
| F-Statistic | 988.5 | *p-*value | 8.197e-16 |  |  |  |

**Centrum Area** as a function of **Centrum Length**, only **Otariidae**

| Variables | Estimate | Std. Error | t-Statistic | Prob | 2.5% | 97.5% |
| --- | --- | --- | --- | --- | --- | --- |
| Intercept | -0.5105 | 0.2441 | -2.091 | 0.0585 | -1.0424 | 0.02150 |
| C Length | 1.8567 | 0.1296 | 14.326 | 6.57e-09 | 1.5743 | 2.1391 |
|  | | | | | | |
| N | 14 | Residual SE | 0.07071 |  |  |  |
| Multiple R^2^ | 0.9448 | Adjusted R^2^ | 0.9402 |  |  |  |
| F-Statistic | 205.2 | *p-*value | 6.567e-09 |  |  |  |

**Neural Spine Height**

**Neural Spine Height** as a function of **Skull Length** and **Family**

| Variables | Estimate | Std. Error | t-Statistic | Prob | 2.5% | 97.5% |
| --- | --- | --- | --- | --- | --- | --- |
| Intercept | -1.5292 | 0.2900 | -5.273 | 1.31e-05 | -2.1232 | -0.9351 |
| SKL Length | 1.4219 | 0.1207 | 11.779 | 2.31e-12 | 1.1747 | 1.6692 |
| Family (Pho) | 0.5152 | 0.3401 | 1.515 | 0.1410 | -0.1815 | 1.2120 |
| SKL : Family | -0.2677 | 0.1409 | -1.900 | 0.0677 | -0.5563 | 0.02087 |
|  |  |  |  |  |  |  |
| N | 32 | Residual SE | 0.04455 |  |  |  |
| Multiple R^2^ | 0.9368 | Adjusted R^2^ | 0.9301 |  |  |  |
| F-Statistic | 138.5 | *p-*value | < 2.2e-16 |  |  |  |

Slopes for Phocidae and Otariidae are not significantly different (*p* = 0.0677)

Note: *Hydrurga leptonyx* (Phocidae) does not have a Centrum Neural Spine Height value – making N = 32.

**Neural Spine Height** as a function of **Skull Length**, only **Phocidae**

| Variables | Estimate | Std. Error | t-Statistic | Prob | 2.5% | 97.5% |
| --- | --- | --- | --- | --- | --- | --- |
| Intercept | -1.0139 | 0.1255 | -8.076 | 4.90e-07 | -1.2801 | -0.7478 |
| SKL | 1.1542 | 0.0513 | 22.497 | 1.55e-13 | 1.0454 | 1.2630 |
|  | | | | | | |
| N | 18 | Residual SE | 0.03146 |  |  |  |
| Multiple R^2^ | 0.9694 | Adjusted R^2^ | 0.9674 |  |  |  |
| F-Statistic | 506.1 | *p-*value | 1.548e-13 |  |  |  |

**Neural Spine Height** as a function of **Skull Length**, only **Otariidae**

| Variables | Estimate | Std. Error | t-Statistic | Prob | 2.5% | 97.5% |
| --- | --- | --- | --- | --- | --- | --- |
| Intercept | -1.5292 | 0.3746 | -4.082 | 0.00152 | -2.3453 | -0.7131 |
| SKL | 1.4219 | 0.1559 | 9.119 | 9.61e-07 | 1.0822 | 1.7617 |
|  | | | | | | |
| N | 14 | Residual SE | 0.05755 |  |  |  |
| Multiple R^2^ | 0.8739 | Adjusted R^2^ | 0.8634 |  |  |  |
| F-Statistic | 83.16 | *p-*value | 9.607e-07 |  |  |  |

**Atlas Transverse Process**

**Atlas Transverse Process** as a function of **Skull Length** and **Family**

| Variables | Estimate | Std. Error | t-Statistic | Prob | 2.5% | 97.5% |
| --- | --- | --- | --- | --- | --- | --- |
| Intercept | -1.2473 | 0.35244 | -3.539 | 0.00138 | -1.9681 | -0.5265 |
| SKL Length | 1.3022 | 0.14672 | 8.876 | 9.19e-10 | 1.0021 | 1.6023 |
| Family (Pho) | -0.12558 | 0.41048 | -0.306 | 0.76184 | -0.9651 | 0.7139 |
| SKL : Family | 0.00771 | 0.16992 | 0.045 | 0.96412 | -0.3398 | 0.3552 |
|  |  |  |  |  |  |  |
| N | 33 | Residual SE | 0.05415 |  |  |  |
| Multiple R^2^ | 0.9162 | Adjusted R^2^ | 0.9075 |  |  |  |
| F-Statistic | 105.6 | *p-*value | 1.039e-15 |  |  |  |

Slopes for Phocidae and Otariidae are not significantly different (*p* = 0.96412)

**Atlas Transverse Process** as a function of **Skull Length**, only **Phocidae**

| Variables | Estimate | Std. Error | t-Statistic | Prob | 2.5% | 97.5% |
| --- | --- | --- | --- | --- | --- | --- |
| Intercept | -1.3729 | 0.12755 | -10.76 | 5.21e-09 | -1.6420 | -1.1038 |
| SKL | 1.3099 | 0.05195 | 25.22 | 6.58e-15 | 1.200 | 1.4195 |
|  | | | | | | |
| N | 19 | Residual SE | 0.03282 |  |  |  |
| Multiple R^2^ | 0.974 | Adjusted R^2^ | 0.9724 |  |  |  |
| F-Statistic | 635.8 | *p-*value | 6.583e-15 |  |  |  |

**Atlas Transverse Process** as a function of **Skull Length**, only **Otariidae**

| Variables | Estimate | Std. Error | t-Statistic | Prob | 2.5% | 97.5% |
| --- | --- | --- | --- | --- | --- | --- |
| Intercept | -1.2473 | 0.4853 | -2.570 | 0.0245 | -2.3047 | -0.1899 |
| SKL | 1.3022 | 0.2020 | 6.445 | 3.18e-05 | 0.8620 | 1.7424 |
|  | | | | | | |
| N | 14 | Residual SE | 0.07456 |  |  |  |
| Multiple R^2^ | 0.7759 | Adjusted R^2^ | 0.7572 |  |  |  |
| F-Statistic | 41.54 | *p-*value | 3.182e-05 |  |  |  |

**Axis Neural Spine Length**

**Axis** **Neural Spine Length** as a function of **Skull Length** and **Family**

| Variables | Estimate | Std. Error | t-Statistic | Prob | 2.5% | 97.5% |
| --- | --- | --- | --- | --- | --- | --- |
| Intercept | -1.8147 | 0.5475 | -3.314 | 0.00247 | -2.9346 | -0.6949 |
| SKL Length | 1.4800 | 0.2279 | 6.493 | 4.16e-07 | 1.01380 | 1.9462 |
| Family (Pho) | 1.0642 | 0.6377 | 1.669 | 0.10592 | -0.2400 | 2.3685 |
| SKL : Family | -0.4757 | 0.2640 | -1.802 | 0.08196 | -1.0156 | 0.06422 |
|  |  |  |  |  |  |  |
| N | 33 | Residual SE | 0.08412 |  |  |  |
| Multiple R^2^ | 0.7749 | Adjusted R^2^ | 0.7516 |  |  |  |
| F-Statistic | 33.28 | *p-*value | 1.591e-09 |  |  |  |

Slopes for Phocidae and Otariidae are not significantly different (*p* = 0.8196)

**Axis** **Neural Spine Length** as a function of **Skull Length**, only **Phocidae**

| Variables | Estimate | Std. Error | t-Statistic | Prob | 2.5% | 97.5% |
| --- | --- | --- | --- | --- | --- | --- |
| Intercept | -0.7505 | 0.3243 | -2.314 | 0.0334 | -1.4348 | -0.06621 |
| SKL | 1.0043 | 0.1321 | 7.603 | 7.25e-07 | 0.7256 | 1.2830 |
|  | | | | | | |
| N | 19 | Residual SE | 0.08346 |  |  |  |
| Multiple R^2^ | 0.7727 | Adjusted R^2^ | 0.7594 |  |  |  |
| F-Statistic | 57.8 | *p-*value | 7.252e-07 |  |  |  |

**Axis** **Neural Spine Length** as a function of **Skull Length**, only **Otariidae**

| Variables | Estimate | Std. Error | t-Statistic | Prob | 2.5% | 97.5% |
| --- | --- | --- | --- | --- | --- | --- |
| Intercept | -1.8147 | 0.5536 | -3.278 | 0.0066 | -3.0209 | -0.6085 |
| SKL | 1.4800 | 0.2305 | 6.422 | 3.29e-05 | 0.9779 | 1.9821 |
|  | | | | | | |
| N | 14 | Residual SE | 0.08505 |  |  |  |
| Multiple R^2^ | 0.7746 | Adjusted R^2^ | 0.7558 |  |  |  |
| F-Statistic | 41.24 | *p-*value | 3.294e-05 |  |  |  |

**Supporting Information: Analysis of Phylogenetic Signal**


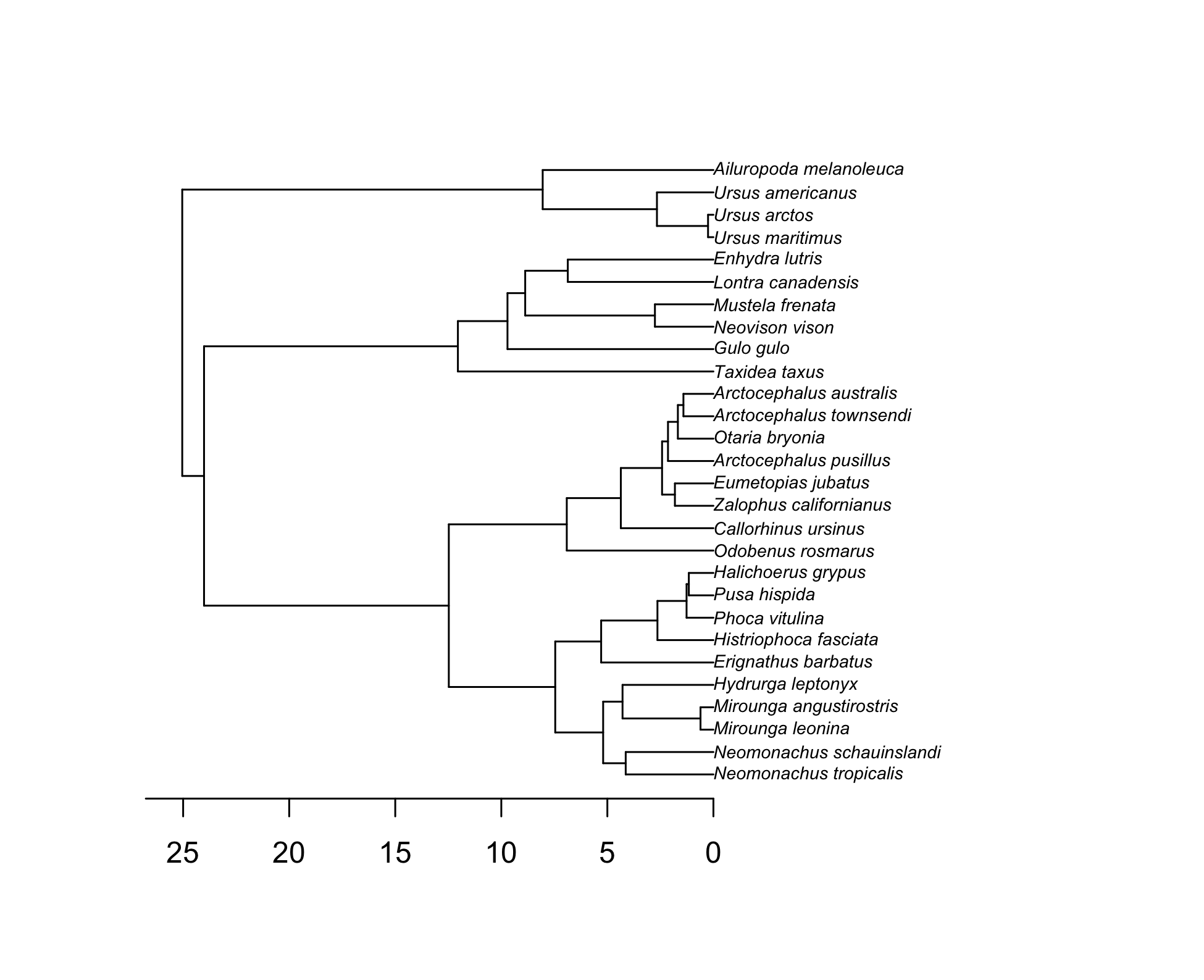


Figure 1: Phylogeny used for the habitat analysis pruned from Upham et al. 2019.


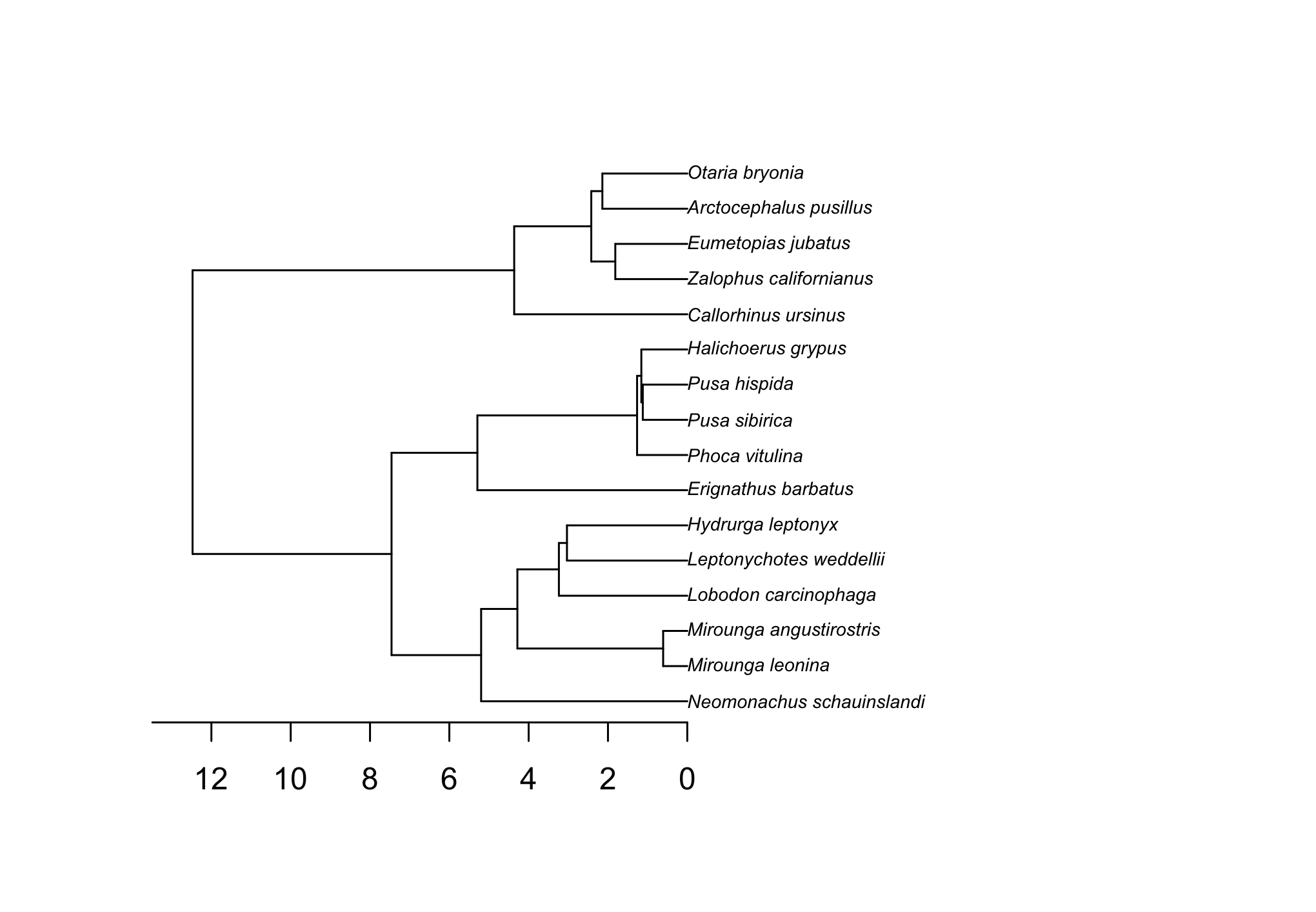


Figure 2: Pinniped Phylogeny pruned from Upham et al. 2019.

Upham, Nathan S., Jacob A. Esselstyn, and Walter Jetz. "Inferring the mammal tree: species-level sets of phylogenies for questions in ecology, evolution, and conservation." *PLoS biology* 17.12 (2019): e3000494.

**Blomberg's K and Pagel’s λ**

From both Blomberg's K and Pagel’s λ, there is no phylogenetic signal found in the residuals. Therefore, there is no need to perform a PGLS to account for the non-independence of the species, and both simple and multiple regressions are appropriate for the dataset. The p-values for nearly all the metrics are not significant. This is due to the low sample size, especially in the analyses using only the pinnipeds. I think we can mention that, but say we still demonstrate that there is low phylogenetic signal, since it is the best we can do with the current dataset.

Table 3. Phylogenetic Signal Results

| Residuals | Blomberg’s K | p-value | Pagel’s λ | logL(λ) | LR(λ=0) | p-value |
| --- | --- | --- | --- | --- | --- | --- |
| Occiput Surface Area as a function of Skull Length | 0.254548 | 0.028 | 7.33137e-05 | 19.6027 | -0.000817594 | 1 |
| Centrum Area as a function of Centrum Length | 0.210227 | 0.724 | 7.33137e-05 | 25.4107 | -0.000776785 | 1 |
| Neural Spine Height as a function of Skull Length | 0.27336 | 0.405 | 7.33137e-05 | 30.3822 | -0.000741749 | 1 |
| Atlas Transverse Process as a function of Skull Length | 0.238571 | 0.609 | 7.33137e-05 | 30.9081 | -0.000809013 | 1 |
| Axis Neural Spine Length as a function of Skull Length | 0.211526 | 0.768 | 7.33137e-05 | 21.1617 | -0.000597685 | 1 |

Multiple Regressions for the Species-level datasets used to calculate Phylogenetic Signal

**Occiput Surface Area** as a function of **Skull Length** and **Habitat Type**

| Variables | Estimate | Std. Error | t-Statistic | Prob | 2.5% | 97.5% |
| --- | --- | --- | --- | --- | --- | --- |
| Intercept | -0.6303 | 0.4823 | -1.307 | 0.204 | -1.6257 | 0.3652 |
| SKL | 1.9502 | 0.2035 | 9.582 | 1.12e-09 | 1.5302 | 2.3703 |
| Habitat (TER) | -0.7913 | 0.5816 | -1.360 | 0.186 | -1.9916 | 0.4091 |
| SKL:Habitat | 0.2650 | 0.2504 | 1.059 | 0.300 | -0.2517 | 0.7817 |
|  | | | | | | |
| N | 28 | Residual SE | 0.1298 |  |  |  |
| Multiple R^2^ | 0.945 | Adjusted R^2^ | 0.9382 |  |  |  |
| F-Statistic | 137.6 | *p-*value | 2.979e-15 |  |  |  |

**Centrum Area** as a function of **Centrum Length** and **Family**

| Variables | Estimate | Std. Error | t-Statistic | Prob | 2.5% | 97.5% |
| --- | --- | --- | --- | --- | --- | --- |
| Intercept | -1.0763 | 0.4786 | -2.249 | 0.046 | -2.1297 | -0.02292 |
| C Length | 2.1623 | 0.2554 | 8.466 | 3.8e-06 | 1.6002 | 2.7244 |
| Family (Pho) | 0.8615 | 0.5048 | 1.707 | 0.116 | -0.2496 | 1.9726 |
| CL: Fam | -0.3928 | 0.2706 | -1.451 | 0.175 | -0.9885 | 0.20294 |
|  | | | | | | |
| N | 15 | Residual SE | 0.05193 |  |  |  |
| Multiple R^2^ | 0.9768 | Adjusted R^2^ | 0.9705 |  |  |  |
| F-Statistic | 154.3 | *p-*value | 2.865e-09 |  |  |  |

**Neural Spine Height** as a function of **Skull Length** and **Family**

| Variables | Estimate | Std. Error | t-Statistic | Prob | 2.5% | 97.5% |
| --- | --- | --- | --- | --- | --- | --- |
| Intercept | -1.01959 | 0.4914 | -2.075 | 0.06227 | -2.1013 | 0.06208 |
| SKL | 1.2074 | 0.2047 | 5.899 | 0.000103 | 0.7569 | 1.6579 |
| Family (Pho) | -0.1136 | 0.5371 | -0.212 | 0.8363 | -1.2957 | 1.0684 |
| SKL:Family | -0.0009908 | 0.2236 | -0.004 | 0.9965 | -0.4930 | 0.4911 |
|  | | | | | | |
| N | 15 | Residual SE | 0.03728 |  |  |  |
| Multiple R^2^ | 0.9569 | Adjusted R^2^ | 0.9451 |  |  |  |
| F-Statistic | 81.32 | *p-*value | 8.603e-08 |  |  |  |

**Atlas Transverse Process** as a function of **Skull Length** and **Family**

| Variables | Estimate | Std. Error | t-Statistic | Prob | 2.5% | 97.5% |
| --- | --- | --- | --- | --- | --- | --- |
| Intercept | -0.5402 | 0.5337 | -1.012 | 0.3314 | -1.7031 | 0.6226 |
| SKL | 1.0051 | 0.2223 | 4.522 | 0.0007 | 0.52078 | 1.4894 |
| Family (Pho) | -0.8711 | 0.5759 | -1.513 | 0.1563 | -2.1257 | 0.3836 |
| SKL:Family | 0.3217 | 0.2395 | 1.343 | 0.2040 | -0.2001 | 0.8435 |
|  | | | | | | |
| N | 16 | Residual SE | 0.04048 |  |  |  |
| Multiple R^2^ | 0.9546 | Adjusted R^2^ | 0.9432 |  |  |  |
| F-Statistic | 84.02 | *p-*value | 2.533e-08 |  |  |  |

**Axis** **Neural Spine Length** as a function of **Skull Length** and **Family**

| Variables | Estimate | Std. Error | t-Statistic | Prob | 2.5% | 97.5% |
| --- | --- | --- | --- | --- | --- | --- |
| Intercept | -0.9107 | 0.9814 | -0.928 | 0.3717 | -3.0491 | 1.2276 |
| SKL | 1.0986 | 0.4087 | 2.688 | 0.0197 | 0.2081 | 1.9892 |
| Family (Pho) | 0.2291 | 1.0589 | 0.216 | 0.8323 | -2.0781 | 2.5364 |
| SKL:Family | -0.1184 | 0.4404 | -0.269 | 0.7926 | -1.07791 | 0.8411 |
|  | | | | | | |
| N | 16 | Residual SE | 0.07444 |  |  |  |
| Multiple R^2^ | 0.7844 | Adjusted R^2^ | 0.7305 |  |  |  |
| F-Statistic | 14.55 | *p-*value | 0.000266 |  |  |  |
